# Molecular Aspects of Seed Development Controlled by Gibberellins and Abscisic Acids

**DOI:** 10.3390/ijms23031876

**Published:** 2022-02-07

**Authors:** Akiko Kozaki, Takuya Aoyanagi

**Affiliations:** 1 Graduate School of Science and Technology, Shizuoka University, Ohya 836, Suruga-ku, Shizuoka 422-8021, Japan; aoyanagi.takuya.17@shizuoka.ac.jp; 2 Course of Bioscience, Department of Science, Graduate School of Integrated Science and Technology, Shizuoka University, Ohya 836, Suruga-ku, Shizuoka 422-8021, Japan; 3 Department of Biological Science, Faculty of Science, Shizuoka University, Ohya 836, Suruga-ku, Shizuoka 422-8021, Japan

**Keywords:** gibberellin (GA), abscisic acid (ABA), seed development, seed maturation

## Abstract

Plants have evolved seeds to permit the survival and dispersion of their lineages by providing nutrition for embryo growth and resistance to unfavorable environmental conditions. Seed formation is a complicated process that can be roughly divided into embryogenesis and the maturation phase, characterized by accumulation of storage compound, acquisition of desiccation tolerance, arrest of growth, and acquisition of dormancy. Concerted regulation of several signaling pathways, including hormonal and metabolic signals and gene networks, is required to accomplish seed formation. Recent studies have identified the major network of genes and hormonal signals in seed development, mainly in maturation. Gibberellin (GA) and abscisic acids (ABA) are recognized as the main hormones that antagonistically regulate seed development and germination. Especially, knowledge of the molecular mechanism of ABA regulation of seed maturation, including regulation of dormancy, accumulation of storage compounds, and desiccation tolerance, has been accumulated. However, the function of ABA and GA during embryogenesis still remains elusive. In this review, we summarize the current understanding of the sophisticated molecular networks of genes and signaling of GA and ABA in the regulation of seed development from embryogenesis to maturation.

## 1. Introduction

Seeds are the products of the evolution of spermatophytes that enable the maintenance and spread of their lineages by providing nutrition for embryo growth and resistance to unfavorable environmental conditions through the state of dormancy. Until the environment is suitable for germination, seeds spend variable lengths of time in the dormancy stage. 

The seed development process can be divided into two main phases, embryogenesis (cell division and morphogenesis) and maturation. Embryogenesis includes the formation and structural development of the mature seeds consisting of an embryo, endosperm, and maternal seed coat. 

As a consequence of complex developmental processes that start from the end of embryogenesis and terminate with the state of dormancy, seed maturation occurs. The maturation stage is characterized by accumulation of storage compound, acquisition of desiccation tolerance, arrest of growth, and entry into dormancy. Seeds can germinate under favorable environmental conditions only after dormancy is broken. 

Complex gene networks regulate seed development and germination, and diverse phytohormones are involved in these processes [1,2]. Gibberellin (GA) and abscisic acids (ABA) are recognized as primary hormones that antagonistically regulate seed development (including dormancy) and germination [2,3]. In early embryogenesis, auxin plays a major role in establishing the embryonic body plan via the effects of apical-basal polarity/pattern formation and vascular development. Together with auxin, cytokinins are linked to growth promotion by cell division, development, and differentiation. Brassinosteroids regulate the ovule number and size and shape of seeds, and also participate in seed germination by antagonizing the inhibitory effect of ABA [1,4].

Until now, impressive progress has been achieved in the understanding of the molecular network regulating the seed development, metabolism, and signaling pathways of ABA and GA in seed maturation and germination. However, the function of ABA and GA during embryogenesis still remains elusive. 

In this review, we summarize the mechanism underlying the regulation of seed development (from embryogenesis to maturation) and the function of the phytohormones GA and ABA in seed development. Since there are several reviews on the function of other phytohormones in seed development in the literature, we focus on these two hormones [1,4,5,6].

## 2. The Level of ABA and GA during Seed Development

### 2.1. ABA Level during Seed Development

**Figure 1:**
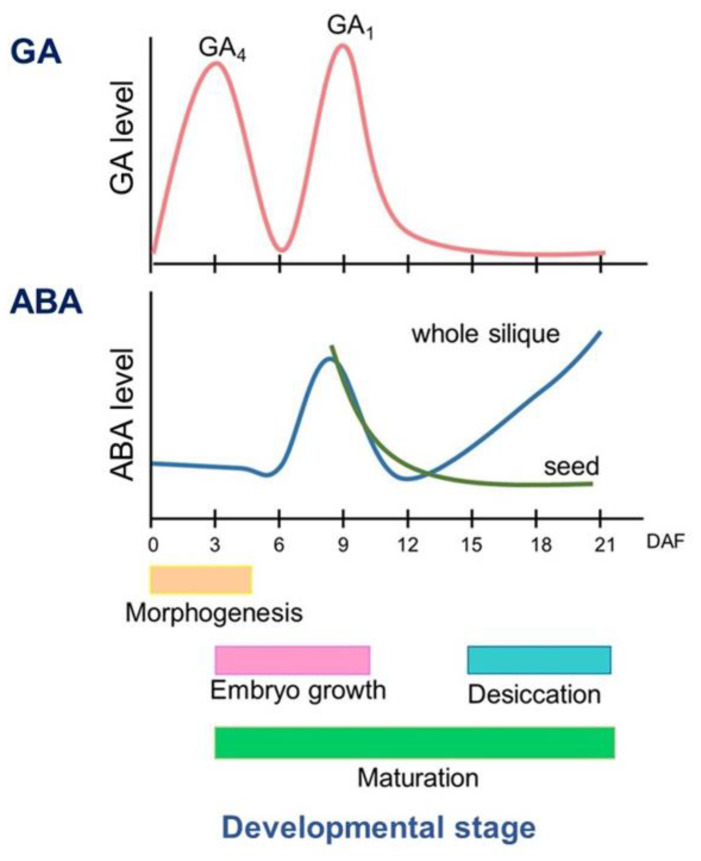
The level of GA and ABA during seed development of *Arabidopsis*. Schematic trend of hormone accumulation during seed development (Based on [7,8,9,10]). DAF: day after flowering.

In seed development in *Arabidopsis*, a peak of ABA level in the whole silique is observed in the middle of development (around nine days after flowering (DAF)), and after 12 DAF, ABA increases until late stage of development (21 DAF) [7,8,9]. ABA was detected mostly in the seeds during the middle stage and in the envelopes during the late stage of maturation [7] (Figure 1). 

It has been demonstrated that nine-cis epoxycarotenoid dioxygenase (NCED) is the key regulatory enzyme in the ABA biosynthetic pathway [11]. Among five *NCED* genes in *Arabidopsis*, *AtNCED6* and *AtNCED9* contribute to a high level of ABA at mid-seed development, while *AtNCED2* and *AtNCED3* contribute to the accumulation of ABA in the later stages of whole silique [9,12]. 

ABA accumulated in both phases is synthesized mainly in zygotic tissues. However, when zygotic tissues, but not maternal tissues, are deficient in ABA, ABA synthesized in maternal tissue is translocated into the embryos of zygotic tissues [7]. The main role of ABA synthesized in zygotic tissues is the induction and/or maintenance of seed dormancy [7,13,14]. On the other hand, maternal ABA affects the thickness of the mucilage layer released from mature seeds on imbibition in *Arabidopsis* [13].

In the seed development of wheat, there are two peaks of ABA level [15,16]. The ABA synthesized during the late phase of seed development (about 35–40 days after pollination (DAP)) is associated with the level of dormancy [15]. On the other hand, rice and triticale have one peak of ABA level in their seed development. In rice seeds, the accumulation of ABA involved in the induction of dormancy occurs during the early and middle stages of seed development (10–20 DAP), earlier than in wheat [17,18]. In triticale grains, peak ABA accumulation was around 35 DAP, before a significant loss of water [19].

Catabolism of ABA occurs by conversion from ABA to phaseic acid (PA), which is catalyzed by a cytochrome P450 monooxygenase (P450) encoded by *CYP707As* [20]. 

### 2.2. ABA Signaling

Three major components are involved in ABA signaling: pyrabactin resistance 1/pyrabactin-like/regulatory components of ABA receptors (PYR/PYL/RCAR), protein phosphatase 2Cs (PP2Cs), and SNF1-related protein kinase 2s (SnRK2s). In the absence of ABA, the activities of SnRK2s is inhibited by PP2Cs through dephosphorylation of their kinase activation loops, while in the presence of ABA, the ABA receptors PYR/PYL/RCAR form a complex with PP2C, which inhibits the phosphatase activity of PP2C and, as a result, SnRK2 is activated [21,22]. The activated form of SnRK2 subsequently activates ABRE-binding protein/ABRE-binding factor (AREB/ABF) transcription factors, which subsequently activate the transcription of ABA-responsive genes [22]. The ubiquitin-proteosome system (UPS) is also involved in ABA signaling. In the absence of ABA, ABA receptors PYR/PYL/RCAR, SnRK2s, and ABREB/ABF transcription factors are degraded via the UPS, which secures the inhibition of the ABA response. On the other hand, PP2C is degraded via the UPS in the presence of ABA leading to the enhancement of the ABA response [23,24].

### 2.3. GA Level during Seed Development

Among more than 130 GAs identified in plants, fungi, and bacteria, only four of them, GA1, GA3, GA4, and GA7, are thought to function as bioactive hormones. And among them, GA1 and GA4 are the major bioactive GAs in many plants including *Arabidopsis*. GA1and GA4 are synthesized via the 13-hydroxy pathway and the non-13-hydroxy pathway, respectively. The latter is the predominant pathway in *Arabidopsis* [25,26].

In *Arabidopsis*, GA4 and GA1 was accumulated in flower buds, flowers, and early developing silique (3 DAF), and in the mid-seed development (around 9 DAF), respectively [7,10,27] (Figure 1). 

The conversion of intermediates to bioactive GAs is catalyzed by two enzymes, GA20-oxidase (GA20ox) and GA3ox in the last steps of the GA biosynthesis. Another enzyme, GA 2-oxidase (GA2ox), catalyzes the conversion of bioactive GAs to inactive catabolites [28]. The level of bioactive GA is controlled primarily by these three enzymes. Bioactive GAs are synthesized in developing seeds by all four *AtGA3ox* and by *AtGA3ox1* in replums and funiculi in developing *Arabidopsis* siliques [27]. In developing pea seeds, *PsGA20ox*s and *PsGA3ox*s were involved in the synthesis of bioactive Gas [29]. 

### 2.4. GA Signaling

GA signaling in plants is induced when bioactive GA is perceived by its receptor GIBBERELLIN INSENSITIVE DWARF1 (GID1) [30,31]. DELLA proteins are negative regulators of GA signaling [32]. When GA binds to GID1, the formation of the GA-GID1-DELLA complex is promoted, and the complex is associated with F-box protein, the central component of SCF^SLY1/GID2^ E3 ubiquitin ligase, which leads to DELLA degradation via the ubiquitin 26S proteasome pathway [33,34,35]. As a result, GA response genes are activated.

## 3. Function of ABA and GA in Seed Development

### 3.1. Function of GA and ABA in Embryogenesis

Embryogenesis starts from a single cell zygote and ends when all embryo structures have been formed. In *Arabidopsis*, embryo development is divided into three phases: the earliest proembryo stage, is characterized by embryo polarity establishment; early embryogenesis is characterized by the embryo morphology shifting from the early globular stage to the heart stage (most of the structures have formed at this stage); and late embryogenesis is characterized by embryo expansion (elongation of cotyledon and axis) and maturation (storage compound accumulation, desiccation, and dormancy) [36]. The last phase, late embryogenesis, corresponds to the early stage of the maturation phase of seed development. 

Several essential genes for embryogenesis, including *YUCCA* (*YUC*) family members, which are auxin biosynthesis genes, and *LEAFY COTYLEDON* genes (*LEC1*, *LEC2,* and *FUSCA3*), have been identified [37,38,39,40,41]. These *LEAFY COTYLEDON* genes also function in the seed maturation stage (described later).

For normal seed development, GAs are required. The evidence that GAs are necessary for seed development has been provided by the analysis of GA-deficient mutant in pea [42,43]. Overexpression of the gene for GA 2-oxidase (GA2ox) from pea in *Arabidopsis* seeds caused seed abortion and inhibition of pollen tube growth, demonstrating that active GAs in the endosperm are essential for normal seed development [44,45]. Similarly, overexpression of GA2ox from tomato in tomato fruit led to the reduction of fruit weight, seed number, and germination rate [46]. 

The maternal tissues, especially the seed coat, play an important role in embryonic development [47,48,49]. The plant proembryo is composed of an embryo-proper domain and a suspensor domain, and the suspensor is the major channel for maternal-to-proembryo communication. The transport of nutrients and signals from the mother to embryo is essential for embryonic development and plant fertility [50,51]. However, the degeneration of the suspensor through programmed cell death (PCD) occurs at a very early stage of embryonic development in plants [52]. In tobacco plant (*Nicotiana tabacum*), the suspensor PCD is established by the antagonistic action of two proteins; a protease inhibitor, cystatin NtCYS, and its target, cathepsin H-like protease NtCP14 [52]. Recently, it has been reported that a DELLA protein, NtCRF (NtCYS regulative factor 1) regulates suspensor PCD in tobacco by promoting the expression of *NtCYS*. GA generated in the micropylar endothelium trigger the suspensor PCD by suppression of *NtCYS* expression via degradation of NtCRF [53]. 

On the other hand, maternal ABA plays a significant role in embryo development and seed maturation in tobacco (*Nicotiana plumbaginifolia*), although it does not affect dormancy induction [54].

### 3.2. Gene Networks in the Maturation Phase

Following the embryogenesis phase, the maturation phase begins. In *Arabidopsis*, the embryo growth phase starts at the torpedo stage (around 7 DAF) and ends when the seed sac is filled with a mature embryo. During the growth phase, the volume ratio of embryo and endosperm is reversed. At the end of the growth phase, the endosperm is reduced to one cell layer while the embryo volume is increased. The cell division of the embryo increases at the beginning of the growth phase and then is arrested by the end of the phase [55]. During the maturation phase, accumulation of seed reserves, maturation and degradation of chloroplasts, acquisition of desiccation tolerance, and dormancy occur before water content decreases and the embryo enters a quiescent state.

In the case of cereals, the endosperm continues to increase the volume to accumulate storage materials and cover other important roles in embryo development and seed organization [1]. 

A complex network of transcription factors regulates seed maturation. Among these, the LAFL regulatory network is the central network. The LAFL genes include the AFL clade of B3 domain plant-specific transcription factors (ALF-B3), FUSCA3 (FUS3), ABA INSENSITIVE 3 (ABI3), LEAFY COTYLEDON 2 (LEC2) [38,39,56], and the HAP3 subunit of the CCAAT-binding transcription factors (CBF or NF-Y), LEC1, and LEC1-LIKE (L1L) [57,58]. Mutation of the LAFL genes affects many aspects of seed maturation: decreased dormancy at maturation [55], reduced expression of seed storage materials [59], reduced desiccation tolerance, and a low level of ABA content [60,61]. The LAFL network regulates several genes involved in modulation of various aspects of plant development besides seed development: genes for zinc finger factor PEI1, APETALA2 (AP2) family factor BABY BOOM (BBM), NAC factor CUP-SHAPED COTYLEDON1 (CUC1), and MADS box factor FLOWERING LOCUS C (FLC) [61].

AFL factors activate the target genes through the RY *cis*-element that is recognized by the B3-DNA binding domain [62,63,64,65,66]. LEC1 and L1L bind to the CCAAT DNA motif as a subunit of the NF-Y complex [57,67]. Genome-wide analysis of LEC1 binding sites in the upstream region of target genes in *Arabidopsis* and soybean revealed that, besides the CCAAT motif, G-Box, ABA-responsive promoter element (ABRE)-like, RY, and BPC1 *cis*-elements were enriched in the promoters of genes regulated during seed maturation, indicating that LEC1 regulates the target genes by interacting with several other kinds of transcription factors [68,69,70]. 

**Figure 2:**
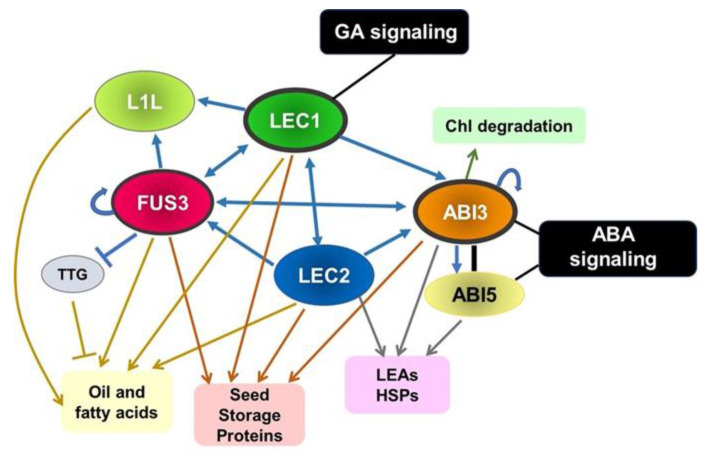
LAFL network regulates seed development. Arrows and blunted lines indicate activation and repression, respectively. Black line between ABI3 and ABI5 indicate the interaction of these proteins. LEC1, LEC2, and FUS3 (surrounded by the thick black line) are involved in acquisition of DT and all LAFL proteins are involved in the regulation of dormancy. LEC1 is related to GA signaling and ABI3 and ABI5 are related to ABA signaling.

Genetic analysis shows that the LAFL genes organize a network with complex mutual interactions among LAFL genes (Figure 2). LEC1 can activate *ABI3*, *FUS3*, and *LEC2* expression, while ectopic expression of *LEC2* can up-regulate *LEC1*, *ABI3*, and *FUS3* [69,71,72]. *ABI3* and *FUS3* positively regulate each other and their own expression [69,73]. Moreover, *L1L* is regulated by FUS3 [74]. A recent ChIP analysis indicated that LEC1 regulates *L1L* [75], whereas FUS3 regulates *LEC1*, *FUS3*, and *ABI3* [76].

In addition to the LAFL genes, ABI5 and ABI5-related bZIP transcription factors (bZIP), which bind to ABRE, are involved in the regulation of seed maturation. ABI5 is a key player in ABA signaling [77]. An important subset of LAFL-regulated genes during seed maturation includes *LATE EMBRYOGENESIS ABUNDANT* (*LEA*) genes, which have both RY and ABRE motifs in their promoters and are regulated by a combination of ABI3 and ABI5-related bZIP transcription factors [78,79]. Therefore, ABA signaling is integrated into the LAFL network by ABI5 and its related bZIP factors via physical interaction with the N-terminal COAR (co-activator/co-repressor) domain of ABI3 [78,79]. ABREs are also found in the promoters of target genes of other LAFL factors, suggesting that other components of the LAFL are potentially co-regulated by ABA [73,75,76].

In *Arabidopsis*, *FUS3* expression is increased by exogenously-introduced ABA [72], and FUS3 induces the increase of ABA [8]. Thus, FUS3 and ABA are positive regulators of each other [41]. Furthermore, the expression of FUS3 was found to be able to be positively regulated by auxin [8]. 

During seed maturation, GA’s level should be down-regulated. GA’s level is regulated by FUS3 and LEC2, which repress the enzymes involved in bioactive GA synthesis [8,80]. 

As mentioned above, LAFL genes play important roles in embryogenesis [70,81]. Recent research showed that GA signaling facilitates embryo development by promoting auxin accumulation in late embryogenesis via *LEC1* in *Arabidopsis*. The GA signaling repressors, DELLAs, interact with LEC1, which promotes the expression of the *YUC* gene that facilitate embryogenesis by promoting the accumulation of auxin. GA triggers the degradation of DELLAs to relieve their repression of LEC1, leading to the activation of genes essential genes for embryogenesis [10]. 

### 3.3. Accumulation of Seed Storage Products

During seed maturation, seed storage compounds needed for germination and initial seedling growth and development, such as seed storage proteins (SSP), lipids, and carbohydrates, are accumulated, and ABA is involved in this process [21,82]. Mutations in ABA signaling, such as *PYL* and *SnRK2*, often exhibit reduced seed storage products [83,84,85,86]. Inactivation of *SnRK2.6* results in reduction of seed oil content, while overexpression of *SnRK2.6* increases overall seed products [84]. *SnRK2s* triple mutant (*snrk2,2/3/6*) and *pyl* duodecuple mutant exhibited lower levels of seed storage products such as 12S globulin [83,85]. The starch biosynthesis in maize and rice is regulated synergistically by sucrose and ABA [87,88,89].

The *LAFL* genes are involved in the regulation of storage material accumulation [90]. LEC1 and FUS3 control the accumulation of ABI3 and function with each other to regulate the accumulation of storage proteins (including *Arabidopsis* 2S albumin storage protein 3 (At2S3) and Cruciferin C (CRC)), anthocyanins synthesis, and accumulation of chlorophyll and lipid during maturation in an ABA-dependent manner [68,72,91,92,93]. LEC1 activates *CRC* as well, via a direct interaction with bZIP67 [74].

FUS3 negatively regulates the expression of *TRANSPARENT TESTA GLABRA1* (*TTG1*), which encodes a transcription factor that suppresses the accumulation of seed storage proteins and oils in *Arabidopsis* [94]. A mutant of *ttg1* is characterized by a dramatic increase in storage reserves, such as oil and SSP [95]. FUS3 may lead to the accumulation of storage reserves by suppressing *TTG1* [94]. FUS3, in combination with LEC2, also induces the expression of *WRINKLED 1* (*WRI1*), which encodes AP2 transcription factor and plays roles in the regulation of sugars and oil content in seeds by increasing the gene expression for fatty acid synthesis and sugar degradation [74,96]. Together with repressing *TTG1* expression and enhancing *WRI1* expression, FUS3 promotes the accumulation of storage oils. This storage oil accumulation is also regulated by *LEC1* and *AFL* genes through activation of *WRI1* [93]. LEC2 regulates oil and protein accumulation by activating the expression of *OLE1*, encoding oleosin and genes encoding 2S and 12S storage proteins [62,63,97,98].

Other factors than *LAFL* genes are also involved in the accumulation of storage materials. bZIP67, together with L1L and NUCLEAR FACTOR-YC2 (NF-YC2), regulate *FATTY ACID DESATURASE 3* (*FAD3*), which functions in the storage of omega-3 fatty acid during maturation [99]. The DOG1-LIKE4 (DOGL4) gene, whose expression is induced by ABA, regulates the expression of some seed storage proteins including CRC, albumins, and oleosins during seed maturation [100].

### 3.4. Desiccation Tolerance and De-Greening

Desiccation tolerance (DT) is an important trait that seeds have to survive prolonged periods until favorable conditions for germination are present for. In many plants, the DT process during seed maturation is intricately linked to loss of chlorophyll (chl), namely de-greening. In terms of commercial products, the presence of chlorophyll in mature seeds can be an undesirable characteristic that can affect seed maturation and quality [60,82]. 

In *Arabidopsis*, the *abi3–6* mutant shows a lack of de-greening, and ABI3 was found to control embryo de-greening through regulating the expression of *STAY GREEN* (*SGR*) genes (*AtSGR1* and *AtSGR2*), which are orthologs of the *SGR* gene encoded by Mendel’s *I* locus [101,102,103]. The seeds of the triple mutant *snrk2.2/3/6* also have greenish-brown seed coats, which indicate that ABA signaling is involved in the de-greening process [83].

*LAFL* genes play important roles in the DT acquisition process. A mutation in *LEC1*, *ABI3*, or *FUS3* drastically affects DT, indicating that all three of these regulators are required to activate DT [104], while a mutation in *LEC2* does not show this effect [69,97]. 

To acquire DT, a set of genes, including genes encoding protective proteins such as LEA [105,106] and HEAT SHOCK PROTEINs (HSPs) [107], and other protective enzymes, compounds, and antioxidants are required [3,108,109,110,111]. LEA proteins are highly hydrophilic glycine-rich proteins that display antioxidant, metal ion binding, membrane and protein stabilization, hydration buffering, and DNA and RNA interaction properties [112,113,114,115].

The expression of the LEA gene is regulated by ABI3 and ABI5 [116,117,118,119]. ABI3 also regulates the expression of seed-specific heat shock factor HSFA9 [120]. LEA and HSP gene expression is increased by DELAY OF GERMINATION (DOG1) through ABI5/ABI3, and enhances the storage of N-rich compounds in the seed, which promotes the seed’s dormancy and viability [117,118,119,121]. 

Although a *lec2* mutant did not show DT reduction [97,122], *LEC2* is involved in DT establishment. LEC2 affects the expression of *LEA*, *EM1*, and *EM6* genes by induction of the expression of the gene for ENHANCED EM LEVEL (EEL) bZIP transcription factor [62], which is a negative regulator of those EM proteins in *Arabidopsis*. EEL competes with a positive regulator of EMS, ABI5, by competing for their promoter sites [118].

In *Medicago truncatula* and pea (*Pisum sativum*), *ABI3*, *ABI4*, and *ABI5* were identified as major hubs to regulate DT acquisition to control genes involved in raffinose family oligosaccharide (RFO) metabolism, LEA proteins synthesis, and photosynthesis associated nuclear genes [106,109,123]. *ABI5* also regulates de-greening and seed longevity in legumes [123].

### 3.5. Induction and Maintenance of Primary Seed Dormancy 

Dormancy, a temporary quiescent state, is the important characteristic of seeds of wild plant species to avoid germination under unfavorable environmental conditions and ensure the initiation of a next generation. Whereas in the case of domesticated species, seeds with fast and uniform germination have been selected for rapid growth to achieve good crop yield. On the other hand, lack of seed dormancy is undesirable because it may cause preharvest sprouting (PHS), a serious problem in cereal crops, and non-dormant mutants can have reduced seed longevity [16,124].

At the end of seed maturation after storage products are synthesized, dehydration starts, and de novo ABA is stored, seed dormancy is achieved [55]. Several pieces of evidence have established that ABA is a key regulator in this process [3,14,124]. Mutation in ABA biosynthesis, sensing, and signaling affect seed dormancy [12,83,85,125,126].

In *Arabidopsis*, mutants of *AtNCED6* and *AtNCED9* show decreased ABA levels and dormancy in mature, dry seeds [12]. Other ABA-deficient mutants, such as *aba1* and *aba2/3*, also show reduced dormancy levels [82,125,126]. In wheat, mutations in the two homologs of *TaABA8’ OH1* (*TaABA8’OH1A* and *TaABA8’OH1D*; *AtCYP707* homolog) resulted in an increase of ABA and an enhanced degree of dormancy [127]. *TsNCED1* is also related to a higher ABA content and higher resistance to PHS [19].

In *Arabidopsis*, AtMYB96 directly activates ABA synthesis genes (*AtNCED2,5,6*, and *9*) and inactivates GA biosynthesis genes (*AtGA3ox1* and *AtGA20ox1*) to induce primary seed dormancy [128]. AtABI4 deepens seed dormancy through direct interaction with promoter regions of *AtNECD6* to increase ABA biosynthesis and, with promoter regions of *AtGA2ox7*, a GA inactivation gene, to inhibit GA accumulation [129,130]. 

A mutation in ABA signaling, such as in the rice *ospyl* septuple and *snrk2.2/3/6* triple mutant, also leads to premature germination in rice and *Arabidopsis* [83,131].

Members of LAFL genes are involved in the achievement of dormancy. Growth arrest of embryo in mature seeds is controlled by *FUS3*, *LEC1*, and *LEC2*, whose mutants all fail in complete cessation of embryo growth and exhibit premature germination [39,98,132].

The maize *Viviparous 1* (*Vp1*) gene, an ortholog of the *ABI3* of *Arabidopsis*, was one of the key ABA signaling components first identified and characterized. A mutation in *Vp1* leads to PHS and disruption of embryo maturation in maize [133,134]. *Vp1* genes of wheat, rice, and sorghum are also associated with the level of dormancy and sensitivity to ABA and PHS [135,136,137]. Members of *LAFL* genes are regulated by the *VP8* encoding of a putative peptidase in maize [138]. The mutations in the *VP8* homolog gene *PLASTOCHRON3/COLIATH* (*PLA3*/*GO*) in rice and *ALTERDMERISTEM PROGRAM 1* (*AMP1*) in *Arabidopsis* show a reduced dormancy phenotype [139].

ABI5 is also important for the induction of dormancy during wheat and pea seed maturation [123,140,141]. In sorghum bicolor, SbABI4 and SbABI5 enhance the transcription of *SbGA2ox3* through directly binding to its promoter, and accordingly prolong seed dormancy [142].

Two major dormancy genes, *DOG1* and *REDUCED DORMANCY 5* (*RDO5*), have been identified that seem to function independently of the plant hormones, including ABA [122,143]. RDO5 is a member of the PP2C protein phosphatase family, but does not show phosphatase activity [143], while DOG1 is a protein of unknown function [122]. Mutations in *DOG1* and *RDO5* completely abolish or reduce seed dormancy, respectively [122,143]. Genetic analysis revealed *DOG1* and ABA are both required for normal seed dormancy [82,122,144]. 

DOG1 interacts with four phosphatases and two of them are belong to clade A of type 2C protein phosphatase, ABA-HYPERSENSITIVE GERMINATION 1 (AHG1), and AHG3. The ABA and DOG1 pathways converge at the level of PP2C phosphatases: DOG1 inhibits AHG1 and AHG3, while ABA inhibits other PP2CAs and AHG3. By inhibiting PP2C phosphatases, ABA and DOG1 promote and maintain dormancy [145,146]. DOG1 is also required for multiple aspects of seed maturation, partially by interfering with ABA signaling components [121].

SEED DORMANCY 4 (OsSDR4) is considered as a regulator involved in seed dormancy with an unknown function in rice [147]. In *Arabidopsis*, SDR4-LIKE (AtSDR4L) regulates dormancy release and germination through regulation of *DOG1* and *RGA-LIKE2* (*RGL2* encoding DELLA protein) in the GA pathway [148]. A recent study speculated that AtODR1 (for reversal of rdo5), an ortholog of OsSDR4, acts together with bHLH57 and functions upstream of *AtNCED6* and *AtNCED9* to control ABA synthesis and seed dormancy in *Arabidopsis* [149].

In addition to gene regulation networks, other regulation, such as protein phosphorylation and chromatin remodeling, is involved in the regulation of dormancy. RAF-like MAPKKKs, RAF10/11 can phosphorylate SnRK2 and ABFs (ABRE binding factors) to influence seed dormancy [150,151]. A member of the histone deacetylation complex in *Arabidopsis*, SIN3-like 1 (SNL1), interacts with HISTONE DEACETYLASE 19 (HDA19) to modulate the ABA signaling pathway to promote seed dormancy [152]. Several regulators, including HISTONE MONOUBIQUITINATION (HUB1: C3HC4-RING finger protein) and REDUCED DORMANCY 2 (RDO2: transcription elongation factor TFIIS), are involved in the regulation of seed dormancy [153,154]. 

## 4. Conclusions and Future Perspectives

ABA and GA play important roles in seed development and germination. Most attention has been paid to the functions of these hormones in the induction, maintenance, and breaking of dormancy and germination [2,3,16,155]. In *Arabidopsis* seed, one of the GA level peaks is at the late stage of embryogenesis as well that of ABA level, during the growth phase of maturation, indicating GA plays an important role at this stage [7,10,27]. However, the detailed function of GA in embryogenesis has remained elusive. A recent finding shed light on the mechanism of GA signaling in the regulation of embryogenesis: GA signaling regulates late embryogenesis via *LEC1* activation [10]. Moreover, GA has been revealed to be a maternal-to-proembryo communication signal to control the embryonic suspensor PCD [53]. 

These recent findings show that DELLA proteins play important roles in the integration of the GA signal with other signals, such as PCD or auxin synthesis [10]. Because DELLA proteins interact with many kinds of proteins and are involved in the various aspects of signal transduction [156,157], novel functions of GA signaling through DELLA proteins in seed development might be found in the future.

Although the importance of maternal ABA in embryogenesis in tobacco was reported [54], the detailed function of ABA in embryogenesis has not been clarified. It has been reported that ABA is required for formation of the somatic embryo, induced by auxin [158]. Auxin promotes the expression of *ABI3,* which induces embryo identity genes through *AUXIN RESPONSE FACTOR* (*ARF*) genes activation [158]. Similarly, auxin controls seed dormancy through stimulation of ABA signaling by inducing *ABI3* expression [159]. Interaction between ABA and auxin signaling has functions in many aspects of plant development [160]. Further research will reveal the detailed functions of ABA in embryogenesis.

In this review, we focus on the function of ABA and GA in seed development. However, besides GA and ABA, there is elaborate crosstalk among phytohormone signaling during seed development. Further research on the crosstalk among signaling of ABA, GA, and other hormones will provide a more complete mechanism of the regulation of seed development.

## Data Availability

Not applicable.
